# Association between *Giardia duodenalis* and Coinfection with Other Diarrhea-Causing Pathogens in India

**DOI:** 10.1155/2014/786480

**Published:** 2014-06-09

**Authors:** Avik K. Mukherjee, Punam Chowdhury, Krishnan Rajendran, Tomoyoshi Nozaki, Sandipan Ganguly

**Affiliations:** ^1^Division of Parasitology, National Institute of Cholera and Enteric Diseases, P-33 CIT Road, Scheme XM, Beliaghata, Kolkata, West Bengal 700010, India; ^2^Division of Data Management, National Institute of Cholera and Enteric Diseases, P-33 CIT Road, Scheme XM, Beliaghata, Kolkata, West Bengal 700010, India; ^3^Department of Parasitology, National Institute of Infectious Diseases, 1-23-1 Toyama, Shinjuku-ku, Tokyo 162-8640, Japan

## Abstract

*Giardia duodenalis*, is often seen as an opportunistic pathogen and one of the major food and waterborne parasites. Some insights of *Giardia* infestation in a diarrhoea-prone population were investigated in the present study. Our primary goal was to understand the interaction of this parasite with other pathogens during infection and to determine some important factors regulating the diarrhoeal disease spectrum of a population. *Giardia* showed a steady rate of occurrence throughout the entire study period with a nonsignificant association with rainfall (*P* > 0.05). Interestingly coinfecting pathogens like *Vibrio cholerae* and rotavirus played a significant (*P* ≤ 0.001) role in the occurrence of this parasite. Moreover, the age distribution of the diarrhoeal cases was very much dependent on the coinfection rate of *Giardia* infection. As per our findings, *Giardia* infection rate seems to play a vital role in regulation of the whole diarrhoeal disease spectrum in this endemic region.

## 1. Introduction 

*Giardia duodenalis* is present worldwide but is more prevalent in developing countries where the lack of sanitation and hygiene awareness is a matter of concern [1, 2]. Considering its high endemicity in some countries, research on* Giardia *is of low priority as the infection it causes is self-limiting, a situation that enhances its propagation. Giardiasis is caused by the protozoan parasite* Giardia duodenalis* [3] which is usually transmitted through ingesting contaminated food and water. A wide variety of pathogens can cause diarrhea, but* G. duodenalis* impacts the economic growth of a country by affecting the Disability Adjusted Life Year (DALY) rates [4]. Giardiasis has much lower mortality rates associated with it than do other diarrheagenic pathogens such as* Vibrio cholerae* or* Shigella* [5]; nevertheless, it may still play an important role in regulating the spectrum of diarrheal diseases in diarrhea-prone regions. The study described herein was designed to survey the prevalence of* G. duodenalis* among diarrheal patients within Kolkata, India. Kolkata is a densely populated city with a variable socioeconomic and climatic background and is frequently affected by outbreaks of diarrheal disease; hence that is why the area was chosen for disease transmission studies [6].

Fecal samples were tested from patients attending the Infectious Diseases and Beliaghata General (IDBG) Hospital in Kolkata city throughout a period of 56 months. These patients only complained of diarrhea. A systemic sampling procedure [7] allowed us to collect enough data to demarcate the catchment areas for diarrhea within the city and to interpret the epidemiological aspects of* Giardia* infestation in an urban region of this developing country.

## 2. Methods

### 2.1. Ethics Statement

This study received ethical clearance from the National Institute of Cholera and Enteric Diseases (NICED) ethical committee, the host institute.

### 2.2. Study Design

The study was performed through collaboration between NICED and IDBG Hospital, Kolkata. IDBG is located within the city of Kolkata and is the largest infectious diseases hospital in India. IDBG treats around 25000 cases of diarrhea every year and most of these patients are residents of the city [6]. Thus, the prevalence of diarrheal diseases in the city can be estimated by surveying IDBG patients. Every fifth patient visiting IDBG who complained of only diarrheal symptoms on two randomly selected days per week was enrolled in the study. The study ran from November 2007 to June 2012. A single fecal sample was sent to the laboratory for analysis by trained healthcare professionals who also obtained the patient's background history via a systematically designed questionnaire. Patient consent for the study was obtained at the same time. The system remained unbiased with regard to sex, age, or other physical factors with nearly proportional distribution of male and female subjects and age ranging from 0 to 60 years in the majority of cases.

### 2.3. Screening for* G. duodenalis* in Stool Samples

*G. duodenalis* was detected in stool samples by using three different procedures. Stool samples were divided into three aliquots immediately after reaching the laboratory. The first aliquot was used for microscopic analysis with iodine wet-mount and trichrome staining [8] after concentration using “Ridley's concentration technique” [9]. The second aliquot was used in an antigen capture enzyme-linked immunoabsorbent assay using a GIARDIAII kit (TechLAB, Blacksburg, VA, USA) as per the manufacturer's protocol. DNA was extracted directly from the third aliquot of each stool sample using a DNA Stool Minikit (Qiagen, USA), according to the manufacturer's protocol. PCR was performed using* G. duodenalis*-specific primers and the DNA extracted by the kit as template following previously published protocols [7, 10]. All of the* G. duodenalis*-positive cases were also investigated for coinfections with other common pathogens as described previously [7]. The bacterial and viral coinfection status of a sample was investigated with assistance from Drs. T. Ramamurthy, T. Krishnan, and M. C. Sarkar in their laboratories at NICED [6].

### 2.4. Statistics and GIS Mapping

Data were entered into the predesigned format of the* pro forma* in the SQL server that has an inbuilt entry validation checking facilitated program by trained data entry professionals. Data were randomly checked and matched for consistency and validity. Edited data were exported and analyzed using SPSS.19.0 and Epi-info 3.5.4 [11].

The inferential age group was explored for* G. duodenalis*-positive cases by multinomial logistic regression [12, 13]. The aim of this was to determine the age groups that were most likely to be infected with* G. duodenalis*. Five age groups were classified, that is, up to 5 years, >5–10 years, >10–20 years, >20–30 years, 30–40 years, and >40 years, and were coded as 1–6, respectively. The relationships between the risk-dependent variable and each of the categorical explanatory variables are shown in Table 1. Infections caused by* G. duodenalis* were classified “1” when the pathogen was present or “2” when absent. The extreme values of the classified age group were fixed as a reference category.

Associations between* G. duodenalis* infection and other variables such as rainfall or coinfection with other pathogens were tested using EpiInfo 3.5.4. Where the presence of* G. duodenalis* was considered an outcome variable, factors like rainfall, overall coinfection, and major coinfection were assigned as dependent variables. Where the* P* value was ≤0.05, this was considered a valid association [14].

**Figure 1:**
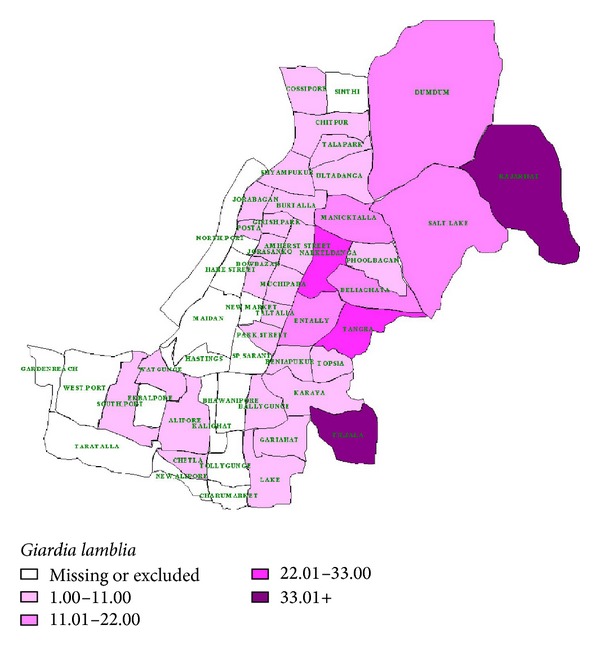
*Giardia duodenalis* distribution area. Map of the study region showing the catchment areas for the* Giardia duodenalis* cases according to our surveillance report (November 2007 to June 2008).

A choropleth map was constructed to display the data from the area where all the positive samples had originated within the city [15]. For this map, the different colors and patterns were combined to depict the different values of the attribute variable associated with each area. Each area is colored according to the category into which its corresponding attribute value had fallen.* G. duodenalis*-positive cases were embedded on the thematic map by the geographical information system (GIS) to visualize the infections. The boundary map shows that the prevalence of* G. duodenalis* was highest in Rajarhat and Tiljala (31.0%), followed by Narkeldanga and Tangra (22–33%), while the values for Dum Dum, Salt Lake, Beliaghata, Maniktala, and Entally regions ranged from 11 to 22 percent (Figure 1).

## 3. Results and Discussion

**Figure 2:**
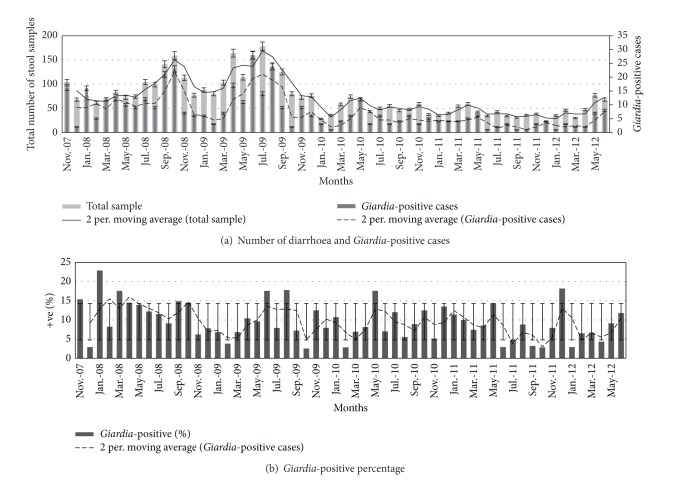
Month-wise distribution of diarrheal cases and* Giardia*-positive cases. (a) The total number of stool samples tested per month throughout the study period and the number of* Giardia*-positive cases recoded for the same period. Note that different* y*-axis scales have been used for clarity. Trend lines representing the sample distribution and* Giardia*-positive cases are similar. (b) Percentage of* Giardia*-positive cases each month. The data show constant deviation and a nonexplanatory trend line.

**Figure 3:**
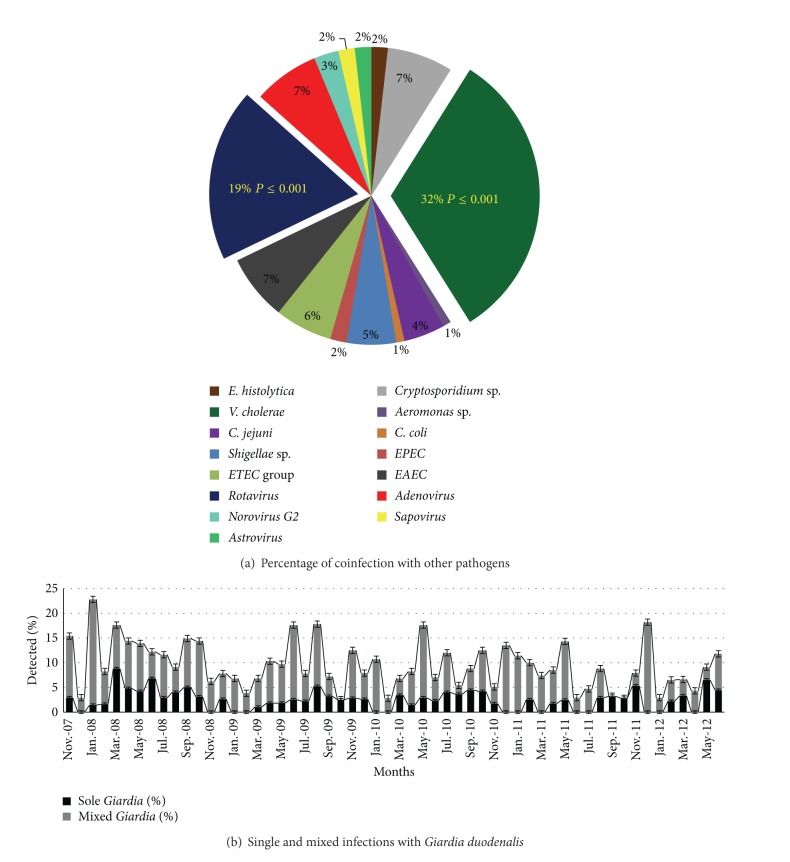
Coinfection of* Giardia duodenalis* with other enteric pathogens. (a) Coinfection of* Giardia* with other pathogens.* Vibrio cholerae* and rotavirus rates are highest and have statistically significant associations (<0.001) with the total number of* Giardia* cases. (b) Monthly prevalence of single and mixed* Giardia duodenalis* infections throughout the study period.

**Figure 4:**
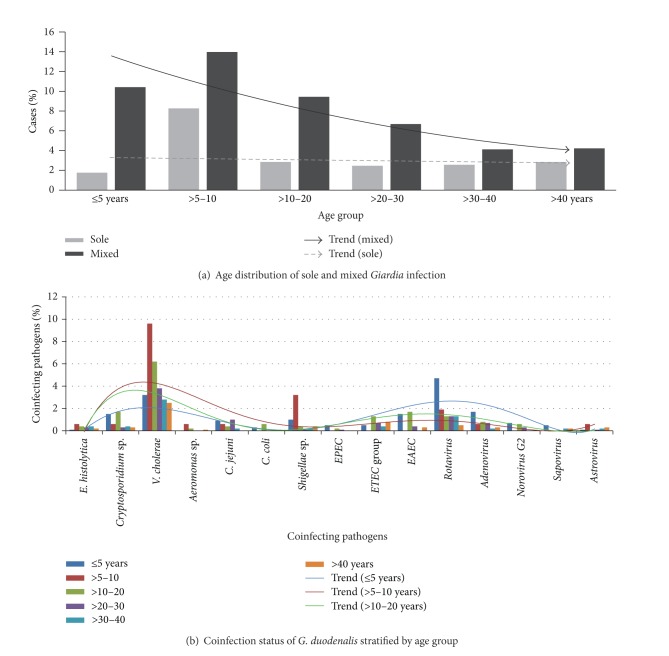
Age-wise distribution of* Giardia* and its relationship with coinfecting pathogens. (a) Distribution of single infections of* Giardia* and mixed infections with other pathogens across six age categories. Note the decreasing slope of the trend line in the older age groups. (b) Age distribution of* Giardia* cases according to their coinfection status with other pathogens. Trend line of ≤5 years shows that coinfection is highest for rotavirus, followed by* Vibrio cholerae,* and for the >5–10 and >10–20 age groups coinfection with* V. cholerae *is higher.

Single stool samples from 4039 diarrheal patients were examined throughout a 56-month period, and 413 (i.e., 10.2%) of them tested positive for* G. duodenalis*. All the data were categorized on a monthly basis to assess any possible seasonality in* Giardia* prevalence. The percentage of* G. duodenalis*-positive cases detected was similar over the entire period with an average detection rate of around 10% each month (Figure 2(a)) and showed a significant correlation with the total number of diarrheal cases in each month (*P* < 0.001). It was evident that the total number of diarrhea cases decreased significantly towards the end of the survey, a trend similar to that observed with* Giardia*-positive cases (Figures 2(a) and 2(b)).* G. duodenalis* showed a statistically significant seasonality and strong association with the total number of diarrheal cases (*P* = 0.001); however, no significant association was found between the numbers of* Giardia*-positive cases and rainfall in the region (*P* > 0.05) (see Supplementary File 1 available online at http://dx.doi.org/10.1155/2014/786480) (Table 1). The number of* Giardia* cases increased during the midsummer to monsoon season (i.e., from May to August). Seventy-four percent of the* Giardia*-positive cases were found to be coinfected with other pathogens, while the remainders were single infections. As per the literature,* Giardia duodenalis* infection may not be associated with diarrhea or related diseases in some cases and rather remain asymptomatic for a long period of time [16, 17], but twenty-six percent of sole infection in the diarrhea patient among the study population demonstrates the symptomatic nature of* Giardia* in this case. Coinfection with* Vibrio cholerae* was the most common (32%), followed by rotavirus (19%) (Figure 3(a)). As all the tests for* Giardia* and other pathogens were conducted over the same set of samples, so the chance of generating data artifact was minimized and the multiple infection could be considered as true coinfection. Infection with* Giardia* showed a strong positive relationship with the presence of other diarrhea-causing pathogens (*P* < 0.001) (Figure 3(b)).* Giardia* infection was very common in the lower age groups and statistically significant associations were found for children ≤5 years and >5–10 years (*P* < 0.001) (Table 2). An age-dependent infection status was also apparent with the two major coinfecting pathogens,* V. cholerae* in the ≤5-year (*P* < 0.001) and rotavirus in >5–10-year (*P* < 0.001) group. Interestingly, coinfections of* Giardia* and other diarrhea-causing pathogens showed a marked decline with increasing age compared with infections with* Giardia *alone (Figure 4(a)).

In spite of observing a trend in the monthly isolation rate for* G. duodenalis*, no seasonality pattern could be inferred from the data; this may be because isolation of the parasite is dependent on the total number of diarrheal cases and this number changes according to the season. However, the steady rates of infection seen in the dry seasons could indicate that* G. duodenalis* is not dependent on rainfall. In this regard, the finding that* Giardia* infections were strongly associated with coinfection (*P* ≤ 0.001) suggests that the parasite derives some advantage from the presence of other diarrhea-causing pathogens in the host, or vice versa. Similarly,* G. duodenalis* was found to be most prevalent in ≤5-year and >5–10-year olds, suggesting that age can be a determining factor for increased susceptibility to Giardiasis. Interestingly, in both of these age groups, coinfections of* Giardia* and rotavirus in children ≤5 years and* Vibrio cholerae* in children above 5–10 years were common (Figure 4(b)). As with previous studies, infection with* V. cholerae* or rotavirus is common in the lower age groups [18] in the study region. This suggests that* Giardia* could in some way take benefit from the major pathogens prevalent in a particular population at a particular time. This could explain the lack of seasonality and steady infection rates among diarrheal cases in regions where* Giardia* is endemic. In the present study, the* G. duodenalis* infection rate is high in the monsoon or postmonsoon period, as did* V. cholerae* and other bacterial pathogens that are associated with water contamination from uncontrolled sewage dispersal in the rainy seasons. However, the rate is also high in the winter, along with coinfecting pathogens such as rotavirus.

## 4. Conclusions

The high rate of* Giardia* infection seen throughout the study period across all climatic conditions and the significant association of* Giardia* with other major pathogens suggest that the parasite may play a role in regulating the spectrum of diarrheal disease in the study area. A statistically significant association with* Vibrio cholerae* and rotavirus across two different seasons suggests that* Giardia* may have evolved to survive in the diarrhea-prone endemic region investigated herein. The opportunistic nature of Giardia is previously considered as an opportunistic pathogen so it can be a major reason for the observation. Otherwise, the coinfection status could be a reason for coexistence of* Giardia* and other pathogens in the infection source, that is, food and water.* Giardia* appears to be maintaining the characteristics of an ideal opportunistic pathogen, resulting in a steady but high prevalence rate in a population and eventually making the population more susceptible to other major diarrheal infections.

## Supplementary Material

It is the regression study showing association among factors like Giardia rate of infection, Total Diarrheal cases, Rainfall, Vibrio cholera (VC) cases, Rotavirus (ROTA) cases with the help of EpiInfo Ver 3.5.4. Program. This particular study was done to understand any possible association between the above mentioned factors. The whole material is sectioned into several parts each starting with a heading like “REGRESS…….=…”. In each section the ‘p' value less than or equals to 0.05 means the association is significant.

## Figures and Tables

**Table 1:** Association between rainfall and *Giardia* prevalence: average seasonal rainfall in the study region (Indian Meteorological Department Database), average *Giardia* detection rates, and the percentage of Giardiasis among all diarrheal cases.

Season	Average rain (mm)	Monthly average *G. duodenalis*-positive cases	Total diarrhea cases	Monthly average *G. duodenalis*-positive (%)
Premonsoon/summer 08	153.4	11	73	15.05
Monsoon 08	1291.7	12.75	103.5	12.02
Postmonsoon 08	70.3	12	110.3	10.1
Winter 09	3.4	4.5	91	4.8
Premonsoon/summer 09	251.8	11.7	123	9.26
Monsoon 09	971.5	18.75	141	13.5
Postmonsoon 09	95.7	5.7	73.3	7.73
Winter 10	16.6	2	34	6.3
Premonsoon/summer 10	143.7	7.3	67	10.83
Monsoon 10	787.4	4	48.25	8.32
Postmonsoon 10	138.8	4.7	48	10.3
Winter 11	5.4	4	37.5	10.7
Premonsoon/summer 11	245.2	5	51.7	10.03
Monsoon 11	1391.6	1.75	35.5	4.87
Postmonsoon 11	29.5	2.7	32	9.6

**Table 2:** Multinomial logistic regression models exploring the significant predominant risk age group for *Giardia duodenalis* infection at IDBG, Kolkata (November 2007–July 2012).

Age in years	*Giardia duodenalis *	*B *	OR (95% CI)	*P* value
≤5 years (*n* = 960)	144	0.56	1.74 (1.29–2.35)	<0.001*
>5–10 years (*n* = 126)	35	1.33	3.79 (2.40–6.00)	<0.001*
>10–20 years (*n* = 375)	60	0.63	1.88 (1.30–2.71)	0.001*
>20–30 (*n* = 551)	64	0.26	1.29 (0.91–1.85)	0.150
>30–40 (*n* = 416)	37	−0.04	0.96 (0.64–1.46)	0.863
>40 years (*n* = 794)	73	*Reference category *		

*n* = sample number.

*Statistically significant.

## Abbreviations

IDBG: Infectious Diseases and Beliaghata General
NICED: National Institute of Cholera and Enteric Diseases
DALY: Disability Adjusted Life Year.
