# Supplementary material for: Association between Giardia duodenalis and Coinfection with Other Diarrhea-Causing Pathogens in India
Source: Biomed Res Int. 2014 Jun 9;2014:786480. doi: 10.1155/2014/786480 (PMC4070398; doi:10.1155/2014/786480)
Supplement: Supplementary file 1 — It is the regression study showing association among factors like Giardia rate of infection, Total Diarrheal cases, Rainfall, Vibrio cholera (VC) cases, Rotavirus (ROTA) cases with the help of EpiInfo Ver 3.5.4. Program. This particular study was done to understand any possible association between the above mentioned factors. The whole material is sectioned into several parts each starting with a heading like “REGRESS…….=…”. In each section the ‘p' value less than or equals to 0.05 means the association is significant. [file 786480.f1.pdf]

Current Data Source: **C:\Users\Avik\Desktop\Giardia Surv Paper\Epiinfo Giardia surv.xls:Sheet1$**
Record Count: **15** *(Deleted Records Excluded)* *Date:* **12-12-2013 17:03:25**

REGRESS PER_Giardia = ACT_RAIN

Linear Regression

Variable	Coefficient	Std Error	F-test	P-Value
**ACT_RAIN**	0.000	0.002	0.0074	0.932738
**CONSTANT**	9.508	0.986	92.9913	0.000000

Correlation Coefficient: r^2= 0.00

Source	df	Sum of Squares	Mean Square	F-statistic
**Regression**	1	0.066	0.066	0.007
**Residuals**	13	115.881	8.914	
**Total**	14	115.947		

REGRESS DIARRHEA = ACT_RAIN

Linear Regression

Variable	Coefficient	Std Error	F-test	P-Value
**ACT_RAIN**	0.014	0.020	0.5292	0.479835
**CONSTANT**	65.911	11.816	31.1160	0.000089

Correlation Coefficient: r^2= 0.04

Source	df	Sum of Squares	Mean Square	F-statistic
**Regression**	1	677.513	677.513	0.529
**Residuals**	13	16642.976	1280.229	
**Total**	14	17320.489		

WRITE	
**COMMANDNAME**	WRITE
**WRITEMODE**	APPEND
**OUTTARGET**	Provider=Microsoft.Jet.OLEDB.4.0;Data Source="C:\Users\Avik\Desktop\Giardia Surv Paper\Mixed assocn data.mdb":Programs
**STATUS**	Export completed successfully, 15 records written.

```
READ {C:\Users\Avik\Desktop\Giardia Surv Paper\Epiinfo Giardia
surv.xls}:[Sheet1$]
REGRESS PER_Giardia=ACT_RAIN
REGRESS DIARRHEA=ACT_RAIN
WRITE APPEND "Epi7" {Provider=Microsoft.Jet.OLEDB.4.0;Data
Source="C:\Users\Avik\Desktop\Giardia Surv Paper\Mixed assocn data.mdb"} :
Programs *
```

REGRESS Total = VC

Linear Regression

Variable	Coefficient	Std Error	F-test	P-Value
**VC**	1.565	0.131	141.7082	0.000000
**CONSTANT**	3.714	0.538	47.6870	0.000000

Correlation Coefficient: r^2= 0.72

Source	df	Sum of Squares	Mean Square	F-statistic
**Regression**	1	1544.551	1544.551	141.708
**Residuals**	54	588.574	10.900	
**Total**	55	2133.125		

REGRESS Total = ROTA

Linear Regression

Variable	Coefficient	Std Error	F-test	P-Value
**ROTA**	1.796	0.361	24.7647	0.000007
**CONSTANT**	4.681	0.881	28.2220	0.000002

Correlation Coefficient: r^2= 0.31

Source	df	Sum of Squares	Mean Square	F-statistic
**Regression**	1	670.684	670.684	24.765
**Residuals**	54	1462.441	27.082	
**Total**	55	2133.125		

REGRESS Total = TOTDIARRHEA

Linear Regression

Variable	Coefficient	Std Error	F-test	P-Value
**TOTDIARRHEA**	0.133	0.013	110.4603	0.000000
**CONSTANT**	-2.238	1.034	4.6892	0.034788

Correlation Coefficient: r^2= 0.67

Source	df	Sum of Squares	Mean Square	F-statistic
**Regression**	1	1432.720	1432.720	110.460
**Residuals**	54	700.405	12.970	
**Total**	55	2133.125		

```
REGRESS PER_Giardia=ACT_RAIN
REGRESS DIARRHEA=ACT_RAIN
WRITE APPEND "Epi7" {Provider=Microsoft.Jet.OLEDB.4.0;Data
Source="C:\Users\Avik\Desktop\Giardia Surv Paper\Mixed assocn data.mdb"} :
Programs *
READ {C:\Users\Avik\Desktop\Giardia Surv Paper\Epiinfo Giardia
surv.xls}:[Sheet2$]
REGRESS PERTotal=PERVC
REGRESS Total=VC

LIST * GRIDTABLE
REGRESS Total=ROTA
REGRESS Total=TOTDIARRHEA
```
